# Evaluation of the Surgical Treatment of Patients over 90 Years Old with Hip Fractures and Their Morbidity and Mortality

**DOI:** 10.1055/s-0044-1779684

**Published:** 2024-03-21

**Authors:** Daniela Burguêz, Rangel Menegatti, Felipe Odeh Susin, Lauro Manoel Etchepare Dornelles, Osvaldo André Serafini

**Affiliations:** 1 Serviço de Ortopedia e Traumatologia, Hospital São Lucas da PUCRS, Porto Alegre, Brasil; 2 Serviço de Ortopedia e Traumatologia, Hospital São Lucas da PUCRS, Porto Alegre, Brasil; 3 Faculdade de Medicina, Pontifícia Universidade Católica do Rio Grande do Sul (PUCRS), Porto Alegre, Brasil

**Keywords:** hip fractures, indicators of morbidity and mortality, nonagenarians

## Abstract

**Objective:** Hip fractures in older adults have the highest impact on the patient's health. These injuries result in many complications, reducing functional capability, quality of life, and life expectancy. This study aimed to provide more epidemiological data on the outcomes of these fractures in nonagenarians from a large city treated at a tertiary hospital.

**Methods:** This study consisted of medical record reviews and interviews.

**Results:** In this study, 76 patients underwent 82 surgeries. The mean age of the patients was 92.5 years. Ninety percent of the subjects were female. The patients spent 10.4 days in hospital. Surgery occurred on average 2.3 days after hospitalization. Regarding fractures, 46 were trochanteric (56%), and 34 affected the femoral neck (41.5%). Forty-one surgeries used the short proximal femoral nail (50%), and 18 were partial hip replacements (22%). During hospitalization, 46 patients (55%) had no complications, excluding episodes of delirium, and seven patients (9%) died. Forty-two subjects completed the one-year postoperative follow-up period, with 56% alive and 44% dead.

**Conclusions:** Treating hip fractures in older patients is challenging. Our goal must focus on helping these subjects receive the quickest and least aggressive treatment possible and start mobilization early. We hope the data presented in this study can lead to a better understanding of the characteristics of our nonagenarian population with hip fractures and seek the best possible treatment for them.

## Introduction

Global population aging increased the number of older patients seeking several medical specialties, including orthopedics. Hip fractures are common in nonagenarian patients. 1 This group of patients reportedly presents higher morbidity and mortality, elevating the costs to the public and private healthcare system. 2

The treatment of choice is virtually always surgical. From the beginning of the 20 ^th^ century onward, several orthopedists in North America and Europe started to indicate surgical treatment instead of conservative treatment, reducing mortality rates. Since then, several devices have been developed for surgical treatment, reducing complications but not changing the mortality rates. 3

Hip fractures in older adults have the highest impact on the patient's health. These injuries result in many complications, reducing functional capability, quality of life, and life expectancy. 4 Some studies show that only around 30% of patients recover their pre-fracture functional status. 5 6

Early mobility is one of the chief measures in preventing post-fracture health problems. 7 Bedridden patients present more infections, especially lung infections. The time from fracture to surgery also seems to improve functional outcomes. 8 Older adults with multiple preoperative comorbidities, regardless of the time until surgery, the type of fracture, and the type of surgery, have worse outcomes. 9

Mortality associated with these types of fractures is high, reaching approximately 20 to 30% within the first year after the event. 10 11 Risk factors associated with worse outcomes include advanced age, male gender, low functional level before fracture, low bone mineral density, previous cognitive dysfunction, low socioeconomic status, and poorly controlled systemic disease. 12 13

To our knowledge, there are no Brazilian studies about clinical outcomes in the specific population of patients over 90 years old. Our study aimed to provide more epidemiological data on the outcomes of these fractures in nonagenarians based on the type of fracture, type of surgery, previous comorbidities, and functional outcomes in the population of a large city treated in a tertiary hospital and followed up for one year after surgery. More reliable data on our population may provide insights to plan more effective treatment measures to reduce complications and morbidity.

## Materials and Methods

This study is cross-sectional, retrospective, descriptive, analytical, and reviewed medical records from patients admitted to the Orthopedics and Traumatology Service of a tertiary hospital in a large city from 2018 to August 2022, aged 90 years or over with a hip fracture undergoing surgical treatment.

The study included all patients operated on after a hip fracture. Subjects under 90 years old, opting for conservative treatment, or who did not want to participate in the study were excluded from the study.

We reviewed the medical records of patients included in the research and interviewed them by telephone to complete the missing data, obtain their informed consent, and gather information at one year of follow-up.

The criteria evaluated included age, gender, previous comorbidities, fracture type, surgical technique, length of stay, time from admission to surgery, postoperative complications, and evolution within one year (mainly morbidity).

The primary outcome was patient mortality during hospitalization and within one year after the procedure. Secondary outcomes included complications, length of stay, time to surgery, and previous comorbidities.

Statistical analysis was univariate. Descriptive statistics characterized the sample as mean, standard deviation, median, interquartile ranges, and absolute and relative frequencies. Bivariate analysis employed the chi-square test to associate independent and dependent variables, adopting a 95% confidence interval and a p-value < 0.05 for statistical significance.

The Scientific Committee of the Medical School approved the research project. The Certificate of Presentation for Ethical Appreciation (CAAE) of this study is 61470022.4.0000.5336.

## Results

Seventy-six subjects underwent 82 surgeries during this period. The mean age of the patients was 92.5 years (range, 90 to 103), and 90% were females ( Table 1 ).

**Table 1:** General features of the population under study

	All patients	Female patients	Male patients
Mean age	92.5 ± 2.3	94.8 ± 3.1	90.5 ± 1.9
Hospitalization days	10.4 ± 3.5	9.8 ± 2.9	10.8 ± 4.8
Days until surgery	2.3 ± 1.2	2.2 ± 1.4	2.5 ± 1.3

**Fig. 1:**
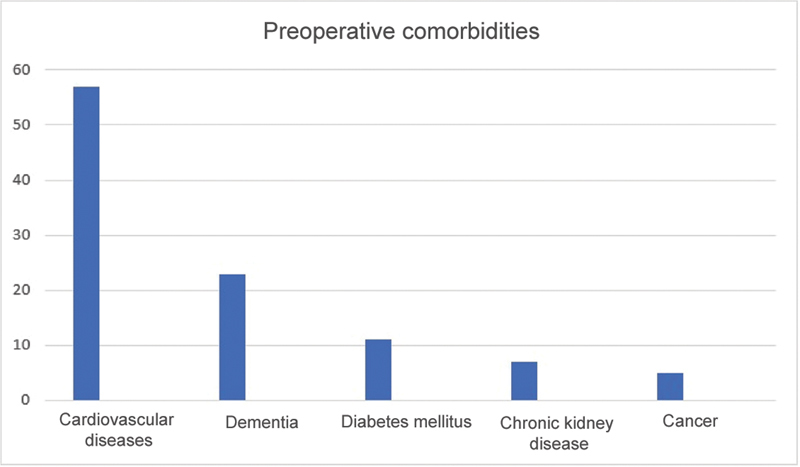
Preoperative comorbidities.

The five most prevalent pre-fracture comorbidities were dementia (23 subjects), cardiovascular diseases (57 patients), diabetes mellitus (11 patients), chronic kidney disease (seven patients), and cancer (five patients) ( Fig. 1 ).

The mean length of stay was 10.4 days (range, 5 to 40 days), and surgery occurred on average 2.3 days (range, 0 to 9 days) after hospitalization ( Table 1 ).

**Fig. 2:**
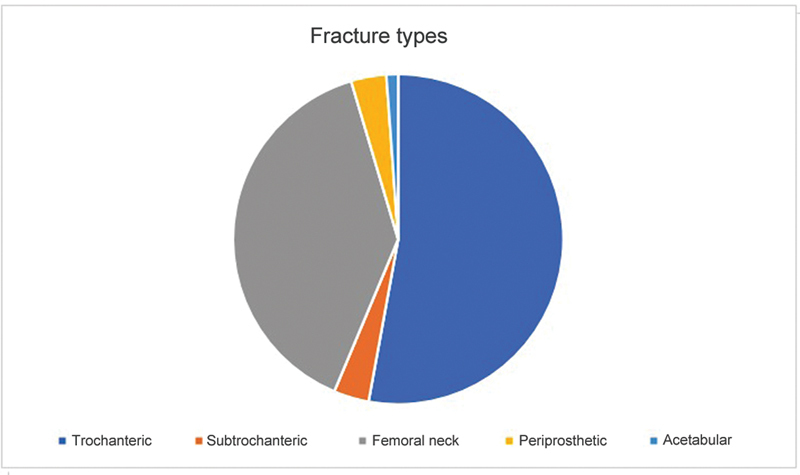
Fracture types.

Regarding the fracture type, 46 were trochanteric, 34 occurred at the femoral neck, three were periprosthetic, three were subtrochanteric, and one was an acetabular fracture ( Fig. 2 ).

**Fig. 3:**
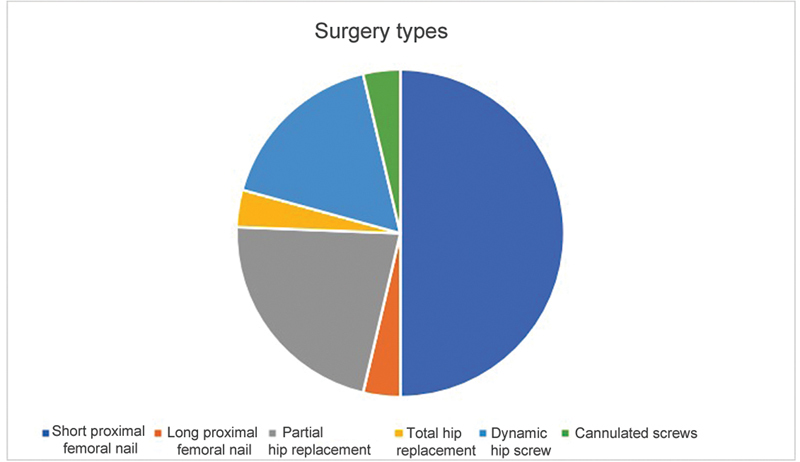
Surgery types.

As for surgical techniques, 41 procedures used a short proximal femoral nail (PFN), 18 were partial hip replacements (PHR), 14 employed femoral dynamic hip screws (DHS), three were osteosyntheses with cannulated screws, three used long PFNs, and three were total hip replacement (THR) ( Fig. 3 ).

**Fig. 4:**
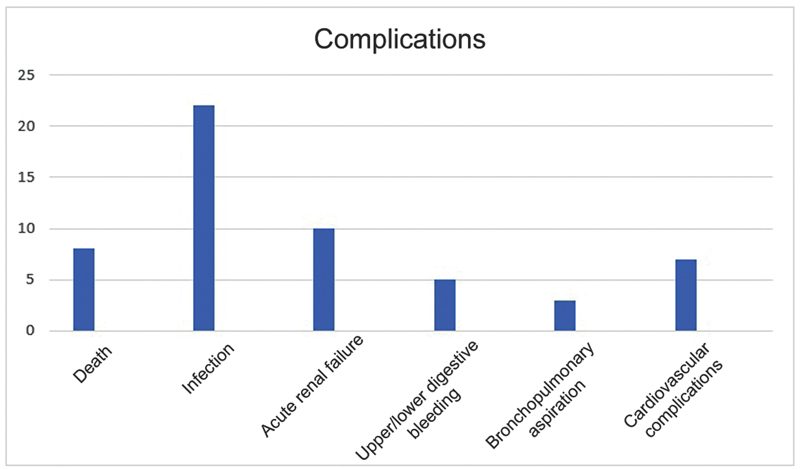
Complications during hospitalization.

During hospitalization, 46 patients (55%) had no complications, excluding episodes of delirium due to its high prevalence in this population, and seven patients (9%) died. Around 30% of all patients experienced an episode of delirium during hospitalization. The most common complication was infection (urinary tract or lung), affecting 22 patients (26.20%). In addition, ten subjects had acute renal failure (ARF), five presented upper or lower digestive bleeding (UDB/LDB), three had bronchopulmonary aspiration, and seven presented cardiovascular complications such as acute myocardial infarction, stroke, and pulmonary embolism ( Fig. 4 ).

**Fig. 5:**
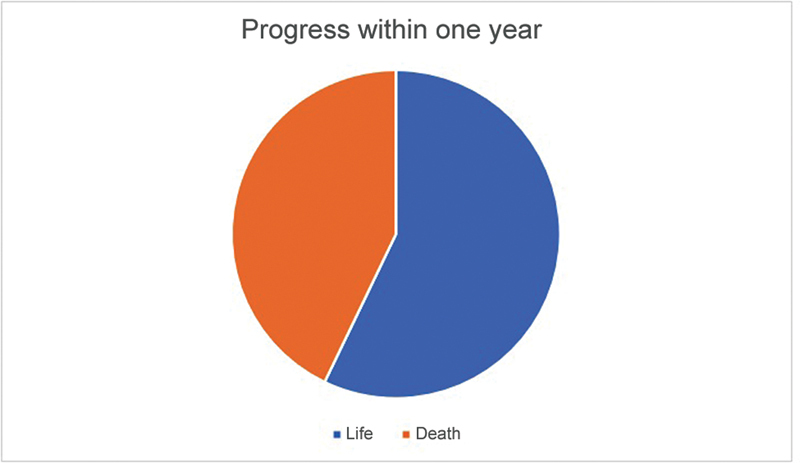
Progress within one year.

Forty-two patients completed one year of postoperative follow-up, with 56% alive and 44% dead (seven patients died during the same hospitalization, and eleven passed away after hospital discharge) ( Fig. 5 ). Another 18 patients also completed one year after surgery, but they were unable to contact or lost at follow-up.

## Discussion

We believe that patients over 90 in the studied region have unique characteristics that differentiate them from other older adults regarding mortality and complications. 14 15 Understanding the demands of this age group is the only way to seek individualized and personalized treatments and improve outcomes.

In our study, the mortality was higher compared with the literature on femoral fractures in people over age 60 (around 25%).

The small sample of male patients hinders gender correlation with worse or better outcomes. Patients with a longer length of stay had worse outcomes and higher mortality (p = 0.027).

The time between hospitalization and surgery does not seem to change the mortality outcome. There was no association between the type of fracture and the type of surgery with patient mortality (p = 0.032). Although the number of trochanteric fractures in older adults is reportedly higher than femoral neck fractures, these figures were similar in our study. 16

Patients with no comorbidities before the fracture had fewer complications during hospitalization, shorter lengths of stay, and lower mortality (p = 0.041). Studies demonstrated that proper disease control before fracture or the lack of comorbidities results in better prognoses regardless of the time from injury to surgery. 17 Consistent with the literature, 18 at least one episode of delirium occurred during hospitalization in 30% of our patients.

Subjects diagnosed with dementia before the fracture did not have worse outcomes or higher mortality than those without dementia. Studies showed that dementia is a risk factor for worse outcomes in patients with hip fractures due to problems during postoperative rehabilitation and early deambulation. However, these studies focused on patients aged over 60. On subjects aged 90 or older, this risk factor seems null. 19 20 21

Patients evaluated one year after the fracture (56% of the 42 subjects completing this follow-up period) reported no major complaints regarding the hip (88% had no complaints while 12% reported mild complaints), and most (92%) resumed the previous walking ability. Among the patients who died within one year after the fracture, seven passed away during the same hospitalization, all due to infection (respiratory or urinary infection progressing to sepsis and death). The remaining 11 patients died after hospital discharge from clinical comorbidities unrelated to the fracture.

Data collection occurred during the coronavirus pandemic, which began in Brazil in March 2019. We observed no decrease in the number of hospitalizations of patients aged 90 or over with hip fractures. There was also no increase in the number of infections affecting this group of patients.

The limitations of our study include the small sample size and the loss at follow-up in one year. We could not contact 10% of patients who completed 12 months of surgery. Another factor that we would have liked to evaluate was deambulation before and after surgery, but this was not feasible. There was missing data in the medical records, and, in some cases, we could not address them in interviews with family members and patients. This outcome is significant since rehabilitation after hip fracture surgery aims at early mobilization and recovery of the independence level before the event.

Treating hip fractures in elderly patients is challenging, even more so in the population over 90 years old. It is a fact that hip fractures reduce the quality of life of these subjects. Our goal must focus on helping these older adults receive the quickest and least aggressive treatment possible and start mobilization early. We hope the data presented in this study can lead to a better understanding of the characteristics of our nonagenarian population with hip fractures and seek the best possible treatment for them.
