# Supplementary material for: Evaluation of the Surgical Treatment of Patients over 90 Years Old with Hip Fractures and Their Morbidity and Mortality
Source: Rev Bras Ortop (Sao Paulo). 2024 Mar 21;59(1):e88–92. doi: 10.1055/s-0044-1779684 (PMC10957275; doi:10.1055/s-0044-1779684)
Supplement: Related texts

# Research article: Avaliação do tratamento cirúrgico de pacientes acima de 90 anos com fraturas de quadril e sua morbimortalidade

**Keywords:** fraturas de quadril, indicadores de morbimortalidade, nonagenários

## Abstract

**Objetivo:** As fraturas de quadril em idosos são as que mais impactam na saúde do paciente e estão associadas a muitas complicações, levando a redução da capacidade funcional, da qualidade de vida e da expectativa de vida. O nosso trabalho visa trazer mais dados epidemiológicos sobre os desfechos dessas fraturas em nonagenários em uma grande cidade atendidos em um hospital terciário.

**Métodos:** O trabalho foi realizado através de revisão de prontuários e entrevistas.

**Resultados:** Foram realizadas 82 cirurgias em 76 pacientes nesse período. A média de idade foi de 92,5 anos, 90% eram mulheres e ficaram 10,4 dias internados. A cirurgia foi realizada em média 2,3 dias após a internação. Do total, 46 fraturas foram trocantéricas (56%) e 34 do colo do fêmur (41,5%). Foram realizadas 41 cirurgias com a técnica da haste cefalomedular curta (50%) e 18 artroplastias parcial de quadril (22%). Durante a internação, 46 pacientes (55%) não apresentaram complicações, excluindo episódios de delirium, e 7 pacientes (9% dos casos) evoluíram para óbito. 42 pacientes já fecharam 1 ano após cirurgia: 56% estão vivos e 44% evoluíram para óbito.

**Conclusões:** O tratamento de fraturas de quadril em pacientes idosos é desafiador. O nosso objetivo deve estar focado em ajudar esses idosos a receber um tratamento rápido e menos agressivo possível e a mobilizar precocemente. Esperamos que, com os dados apresentados nesse trabalho, possamos entender melhor acerca das características da nossa população nonagenária vítimas de fratura de quadril e buscar o melhor tratamento possível para esses pacientes.

## Introdução

O envelhecimento da população mundial trouxe cada vez mais pacientes mais velhos para diversas especialidades, incluindo a ortopedia. As fraturas de quadril são comuns em pacientes nonagenários. 1 Sabe-se que esse grupo de pacientes tem associação com grande morbimortalidade, trazendo altos custos para o sistema de saúde, tanto público como privado. 2

O tratamento de escolha quase sempre é cirúrgico. Desde o início do século XX, vários ortopedistas na América do Norte e Europa começaram a propor o tratamento cirúrgico ao invés do conservador. Se observou redução da mortalidade. Desde então, vários dispositivos foram desenvolvidos para o tratamento cirúrgico, sendo aprimorados para suas complicações, porém sem alterar a mortalidade desses pacientes. 3

As fraturas de quadril em pacientes idosos são as que mais impactam na saúde do paciente e estão associadas a muitas complicações, levando a redução da capacidade funcional, da qualidade de vida e da expectativa de vida. 4 Alguns estudos mostram que apenas cerca de 30% dos pacientes recuperam seu quadro funcional prévio a fratura. 5 6

A mobilidade precoce é uma das principais medidas na prevenção de agravos de saúde pós-fratura. 7 Pacientes acamados são acometidos por mais infecções, principalmente pulmonares. O tempo entre a fratura e a cirurgia também parece melhorar os desfechos funcionais. 8 Idosos com múltiplas comorbidades prévias a fratura, independente do tempo para cirurgia, do tipo de fratura e do tipo de cirurgia proposta, apresentam desfechos piores. 9

A mortalidade associada a esses tipos de fraturas são altas, chegando a cerca de 20-30% dentro do primeiro ano após o evento. 10 11 Os fatores de risco associados a piores desfechos são idade avançada, sexo masculino, baixo nível funcional prévio a fratura, baixa densidade mineral óssea, disfunção cognitiva prévia, baixo nível socioeconômico e doença sistêmica mal controlada. 12 13

Não foram encontrados estudos no Brasil para desfechos clínicos na população específica de pacientes acima de 90 anos. O nosso projeto visa trazer mais dados epidemiológicos sobre os desfechos dessas fraturas em nonagenários baseado no tipo de fratura, tipo de cirurgia, comorbidades prévias e desfechos funcionais na população de uma cidade grande atendida em um hospital terciário em até 1 ano após a cirurgia. Assim, de posse de dados mais fidedignos da nossa população, poderemos planejar medidas mais efetivas para tratamento, tentando reduzir complicações e morbidade.

## Materiais e Métodos

Trata-se de um estudo transversal descritivo e analítico retrospectivo. Foram avaliados os prontuários dos pacientes internados pelo Serviço de Ortopedia e Traumatologia de um hospital terciário de uma cidade grande de 2018 até agosto de 2022 com idade igual ou superior a 90 anos com fratura de quadril que foi optado pelo tratamento cirúrgico.

Foram incluídos todos os pacientes submetidos a cirurgia após fratura de quadril e que tenham 90 anos ou mais. Foram excluídos no estudo pacientes com menos de 90 anos ou que tenha se optado pelo tratamento conservador da fratura ou que não quiseram participar do estudo.

Foi realizada revisão de prontuário dos pacientes inclusos na pesquisa e entrevista por telefone dos dados faltantes, bem como coleta do Termo de Consentimento Livre e Esclarecido, e acompanhamento por 1 ano.

Foram avaliados os seguintes critérios: idade, sexo, comorbidades prévias, tipo de fratura, técnica cirúrgica escolhida, tempo de internação, tempo do momento de internação até a cirurgia, complicações pós-operatórias e evolução em 1 ano (principalmente morbidade).

O desfecho primário avaliado foi a mortalidade dos pacientes durante internação e dentro de 1 ano do procedimento. Os desfechos secundários foram complicações, dias de internação, tempo até a cirurgia e comorbidades prévias.

A análise estatística foi realizada de forma univariada. A estatística descritiva foi utilizada para caracterização da amostra através da média, desvio-padrão, mediana, amplitude interquartil e frequências absolutas e relativas. Para análise bivariada, foi utilizado o teste do qui-quadrado para associação de variáveis independentes e dependentes, sendo adotado o intervalo de confiança de 95% e o valor p < 0,05 para significância estatística.

O presente projeto de pesquisa foi aprovado pela Comissão Científica da Faculdade de Medicina e Hospital na qual o estudo foi realizado. O Certificado de Apresentação para Apreciação Ética (CAAE) desse trabalho é 61470022.4.0000.5336.

## Resultados

Foram realizadas 82 cirurgias em 76 pacientes nesse período. A média de idade dos pacientes foi de 92,5 anos (90-103 anos) e 90% eram do sexo feminino ( Tabela 1 ).

**Tabela 1:** Características gerais da população estudada

	Todos	Mulheres	Homens
Idade média	92,5 ± 2,3	94,8 ÷ 3,1	90,5 ± 1,9
Dias internados	10,4 ± 3,5	9,8 ± 2,9	10,8 ± 4,8
Dias para cirurgia	2,3 ± 1,2	2,2 ± 1,4	2,5 ± 1,3

De comorbidades prévias a fratura, apresentamos as 5 mais prevalentes. Vinte e três pacientes apresentavam demência, 57 doenças cardiovasculares, 11 diabetes mellitus, 7 doenças renal crônica e 5 cânceres ( Fig. 1 ).

**Fig. 1:** Comorbidades prévias a cirurgia.

Nossos pacientes ficaram em média 10,4 dias internados (5-40 dias) e a cirurgia foi realizada em média 2,3 dias (0-9 dias) após a internação ( Tabela 1 ).

Do total, 46 fraturas foram trocantéricas, 34 do colo do fêmur, 3 periprotéticas, 3 subtrocantéricas e 1 de acetábulo ( Fig. 2 ).

**Fig. 2:** Tipos de fratura.

Foram realizados 41 cirurgias com a técnica da haste cefalomedular (PFN, em inglês) curta, 18 artroplastias parcial de quadril (APQ), 14 placa com parafuso dinâmico (DHS, em inglês) de fêmur, 3 osteossíntese com parafusos canulados, 3 PFN longo e 3 artroplastias totais de quadril (ATQ) ( Fig. 3 ).

**Fig. 3:** Tipos de cirurgia.

Durante a internação, 46 pacientes (55%) não apresentaram complicações, excluindo episódios de delirium devido a sua alta prevalência nessa população, e 7 pacientes (9% dos casos) evoluíram para óbito. Cerca de 30% de todos os pacientes apresentaram algum episódio de delirium durante a internação. A complicação mais comum foi infecção (do trato urinário ou pulmonar) em 22 pacientes (26,20%). Além disso, 10 pacientes apresentaram insuficiência renal aguda (IRA), 5 hemorragia digestiva alta ou baixa (HDA/HDB), 3 broncoaspiração e 7 complicações cardiovasculares como infarto agudo do miocárdio, acidente vascularcerebral e embolia pulmonar ( Fig. 4 ).

**Fig. 4:** Complicações durante internação.

Quarenta e dois pacientes já fecharam um ano após cirurgia: 56% estão vivos e 44% evoluíram para óbito (7 pacientes faleceram na mesma internação, 11 após a alta hospitalar) ( Fig. 5 ). Outros 18 pacientes também passaram um ano após a cirurgia, porém não conseguimos contato com eles nem mantiveram acompanhamento.

**Fig. 5:** Evolução em 1 ano.

## Discussão

Acreditamos que pacientes com mais de 90 anos da região estudada apresentam características únicas que os diferem dos demais idosos em relação a mortalidade e complicações. 14 15 Somente entendendo as demandas dessa faixa etária é possível buscar um tratamento individualizado e personalizado que melhorem os desfechos.

A mortalidade encontrada no nosso estudo é maior do que aquela apresentada na literatura para fraturas de fêmur em pessoas acima de 60 anos (cerca de 25%).

Devido a pequena amostra de pacientes do sexo masculino, não é possível correlacionar piores ou melhores desfechos em relação ao gênero. Pacientes que tiveram maior tempo de internação apresentaram piores desfechos e maior associação com mortalidade (p = 0,027).

O tempo entre a internação e a cirurgia não parece modificar o desfecho mortalidade. Não houve associação entre o tipo de fratura e o tipo de cirurgia com a mortalidade dos pacientes (p = 0,032). Sabe-se que fraturas trocantéricas afetam mais pacientes mais idosos do que fraturas do colo do fêmur, porém, na nossa amostra, esse número foi similar. 16

Pacientes sem comorbidades prévias a fratura apresentaram menos complicações durante a internação, menor tempo de internação e menor mortalidade (p = 0,041). Estudos vem demonstrando que o bom controle de doenças prévias a fratura ou a ausência de comorbidades apresentam melhores prognósticos independentemente do tempo entre a fratura e a cirurgia. 17 Pelo menos um episódio de delirium ocorreu em 30% da população estudada durante a internação, número semelhante ao encontrado na literatura. 18

Pacientes com diagnóstico de demência prévia a fratura não apresentaram piores desfechos nem maior mortalidade em relação ao grupo de pacientes sem demência. Estudos mostram que a demência é um fator de risco para piores desfechos em pacientes que sofrem fraturas de quadril devido a dificuldade na reabilitação e deambulação precoce após a cirurgia. Porém, esses estudos foram feitos em pacientes com mais de 60 anos. Em um grupo com idade igual ou maior de 90 anos, esse fator de risco parece se anular. 19 20 21

Os pacientes que foram avaliados em 1 ano após a fratura (56% dos 42 pacientes que fecharam um ano) não relatavam queixas em relação ao quadril (88% sem nenhuma queixa, 12% com queixas leves) e a maioria havia recuperado a capacidade de deambular prévia (92%). Dos pacientes que faleceram dentro de 1 ano após a fratura, 7 tiveram óbito na mesma internação, todos por infecção (infecção respiratória ou urinária com evolução para sepse e óbito). Os outros 11 pacientes morreram após a alta hospitalar de comorbidades clínicas não relacionadas à fratura.

Os dados foram coletados também no período da pandemia pelo coronavírus, que se iniciou em março de 2019 no Brasil. O que se pode observar foi que não houve diminuição do número de internações em pacientes com 90 anos ou mais com fraturas de quadril. Também não houve aumento no número de infecções que acometem essa parcela de pacientes.

As limitações do nosso estudo são a amostra pequena e a perda de seguimento em 1 ano. Não foi possível contato com 10% dos pacientes que fecharam 12 meses de cirurgia. Outro fator que gostaríamos de ter avaliado porém não foi possível foi a deambulação prévia e pós cirurgia. Faltaram dados no prontuário e/ou não foi possível qualificar via entrevista com os familiares e os pacientes. Esse desfecho é importante visto que a cirurgia pós fratura de quadril visa a mobilização precoce e a recuperação da independência prévia ao evento.

O tratamento de fraturas de quadril em pacientes idosos é desafiador. Na população com mais de 90 anos, parece ser ainda mais difícil. É fato consolidado que fraturas de quadril reduzem a qualidade de vida desses pacientes. O nosso objetivo deve estar focado em ajudar esses idosos a receber um tratamento rápido e menos agressivo possível e a mobilizar precocemente. Esperamos que, com os dados apresentados nesse trabalho, possamos entender melhor acerca das características da nossa população nonagenária vítimas de fratura de quadril e buscar o melhor tratamento possível para esses pacientes.
